# A Federated Design for a Neurobiological Simulation Engine: The CBI Federated Software Architecture

**DOI:** 10.1371/journal.pone.0028956

**Published:** 2012-01-05

**Authors:** Hugo Cornelis, Allan D. Coop, James M. Bower

**Affiliations:** 1 Cornelis H. Research Imaging Institute, University of Texas Health Science Center at San Antonio, San Antonio, Texas, United States of America; 2 Coop A. D. Department of Epidemiology and Biostatistics, University of Texas Health Science Center at San Antonio, San Antonio, Texas, United States of America; 3 Bower J. M. Research Imaging Institute, University of Texas Health Science Center at San Antonio, San Antonio, Texas, United States of America; University of Alberta, Canada

## Abstract

Simulator interoperability and extensibility has become a growing requirement in computational biology. To address this, we have developed a federated software architecture. It is federated by its union of independent disparate systems under a single cohesive view, provides interoperability through its capability to communicate, execute programs, or transfer data among different independent applications, and supports extensibility by enabling simulator expansion or enhancement without the need for major changes to system infrastructure. Historically, simulator interoperability has relied on development of declarative markup languages such as the neuron modeling language NeuroML, while simulator extension typically occurred through modification of existing functionality. The software architecture we describe here allows for both these approaches. However, it is designed to support alternative paradigms of interoperability and extensibility through the provision of logical relationships and defined application programming interfaces. They allow any appropriately configured component or software application to be incorporated into a simulator. The architecture defines independent functional modules that run stand-alone. They are arranged in logical layers that naturally correspond to the occurrence of high-level data (biological concepts) versus low-level data (numerical values) and distinguish data from control functions. The modular nature of the architecture and its independence from a given technology facilitates communication about similar concepts and functions for both users and developers. It provides several advantages for multiple independent contributions to software development. Importantly, these include: (1) Reduction in complexity of individual simulator components when compared to the complexity of a complete simulator, (2) Documentation of individual components in terms of their inputs and outputs, (3) Easy removal or replacement of unnecessary or obsoleted components, (4) Stand-alone testing of components, and (5) Clear delineation of the development scope of new components.

## Introduction

The application of mathematical methods to modeling and quantification in neurophysiology can be traced to the Lapicque model of a neuron introduced over a century ago [1], the empirical description of action potential generation and propagation [2], the application of cable theory to the modeling of dendritic electrophysiology [3], and the recognition that different levels of analysis could be employed to understand brain function [4]. Although, a hand cranked calculator was employed to verify the original integration of the action potential, it was not until mathematical approaches based on cable theory were developed to model dendritic properties and function in the late 1950s, that digital computers became a necessary tool for modeling studies [5]. It took a further quarter century for the interdisciplinary field that links neuroscience, cognitive science, electrical engineering, computer science, physics, and mathematics to be named and thus give birth to computational neuroscience [6].

Historically, the development of neuronal simulation software for the construction of morphologically detailed neuron models and small networks was instigated by research projects that specifically addressed complementary technical and scientific questions [7]. For example, one widely used application is NEURON (http://www.neuron.yale.edu/neuron/, [8]). It grew from the identification of numerical techniques that greatly improved the efficient computation and accuracy of the solution to the cable equations employed to model electrical activity in branched dendrites [9], [10]. Another widely used simulation platform is GENESIS (http://genesis-sim.org, [11]). From conception, it has been a more generalized simulator and was initially employed to model at the single cell level neural oscillations in piriform [12] and cerebral cortex [13].

These software systems have both been highly successful and have continued to grow in complexity through cycles of research project extension (see for example parallel NEURON [14] and PGENESIS [15]). However, after more than twenty years of extending their functionality, usually by the direct incorporation of source code into the core of the simulator, code structures have become so complicated that it is now increasingly difficult, if not impossible, to easily continue this process. The resulting stand-alone applications have become *monolithic* with their life cycles inevitably moving from extension to maintenance. (Note: *italicized* text indicates the first appearance of a technical term defined in Materials and Methods. Typewriter text indicates the name of a software *module* described in Results.) A significant consequence of this process is that the development of an optimized simulation can be a considerable challenge for the neuroscientist unfamiliar with the underlying mathematical and computational theory.

One response to the cumbersome nature of *monolithic software* applications has been the development of specialized simulators capable of modeling different levels of biological detail. For example, NEST (http://www.nestinitiative.org/index.php/About_Us, [16]) simulates large structured network systems, HHsim (http://www.cs.cmu.edu/dst/HHsim/, [17]) provides a graphical environment for the detailed exploration of a section of excitable membrane via the Hodgkin-Huxley equation formalism, COPASSI (http://www.copasi.org/tiki-view_articles.php, [18]) is a SBML [19] enabled application for simulation and analysis of sub-cellular biochemical networks, and MCell (http://www.mcell.cnl.salk.edu/, [20]) uses Monte Carlo algorithms to track the stochastic diffusion of individual molecules.

The rapidly growing diversity of modeling environments and tools raises significant issues surrounding the reproducibility of results from different simulators. This is not a trivial problem. When combined with the current laxity in reporting model and simulation details and the general lack of independent validation of computationally generated results, the credibility of research in computational neuroscience is frequently compromised. In principle, any finding should be independently reproduced prior to being accepted as a genuine contribution to the body of scientific knowledge (see [21], [22]).

Problems of incremental model extension, incomplete model specification, and reproducibility of results, have resulted in the idea of the “interoperability” of neuroscience modeling software. Interoperability has been defined as “all mechanisms that allow two or more simulators to use the same model description or to collaborate by evaluating different parts of a large neural model” [21]. Various forms of interoperability are possible and two broad types have recently been identified [21]. Type 1 interoperability is defined as the development of portable model description standards such that models built for one simulator can be run on another, i.e. through the adoption of common simulation languages such as SBML (http://sbml.org/, [19]), NeuroML (http://www.neuroml.org/, [23]), or NineML (http://software.incf.org/software/nineml). Type 2 is defined as run-time interoperability, where different simulators operating on different domains interoperate at run-time either by direct coupling via simulator script languages (e.g. pyMOOSE [24]) http://moose.ncbs.res.in/component/option,com_wrapper/Itemid,86/; MUSIC [25]), indirect coupling via interpreted languages (e.g. PyNN http://neuralensemble.org/trac/PyNN, [26]), or coupling via object oriented frameworks (e.g. Catacomb2 http://www.compneuro.org/catacomb/ccmb_help/index.html, [27]).

Here we introduce a new class of simulator architecture, the Computational Biology Initiative federated software architecture (for convenience referred to here as the CBI architecture). It takes its name from the Computational Biology Initiative at the University of Texas at San Antonio, where development was first initiated. It has been fully implemented as the basis of the recently reconfigured GENESIS (see [28]). As we now show, it is a *software architecture* that transparently supports both interoperability and “extensibility” for model building, simulation, and result analysis.

## Results

A biological system can be characterized as rich, complex, and multi-dimensional. These characteristics distinguish it from the mathematical representations and data formats employed by a computer-based model. Specifically, the dynamical properties of a biological system do not map easily to the logical principles and mathematical constructs used by software implementations [29]. This has the important consequence that current model representation technology employed by simulators typically exposes mathematical details and peripheral control code to a user that is unrelated to the biology that it aims to represent (see Supplementary Material–S1, S2 for examples from GENESIS 2; S3, S4 for examples from NEURON).

To address these issues, we developed the CBI architecture, a meta-framework for software development that defines a fully functioning simulator. The architecture is the principled result of a bottom-up restructuring of the source code of the GENESIS 2 neural simulation system. It was developed on the basis of our understanding of a general scientific workflow, referred to as an *ideal user workflow*, and an analytic method known as a *separation of concerns*. The software modules resulting from this analysis are described and a brief overview of the structural and functional relationships of the CBI architecture is then given.

### The Scientific Workflow

**Figure 1:**
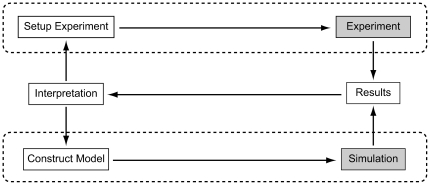
Data flows in science. Conducting experiments and running simulations are two iterative processes indicated by the upper and lower dashed outlines. They are connected by an interposed feedback loop that uses the iterative interpretation of results to design new experimental setups and model constructions.

The relationship in computational biology between the activities involved in conducting an experiment and running a simulation is illustrated in Figure 1. These two iterative processes are connected by a feedback loop that employs interpretation of results as an iterator to design new experimental setups and model constructions.

From this perspective, simulation provides a framework to organize our understanding of biological systems. The CBI architecture is designed to support the lower loop within the illustrated scheme and was developed in an effort to resolve the complexities associated with continual addition of functionality to simulators. Ultimately, simulators become monolithic and it is increasingly difficult for users and developers to maintain and extend them, with the logical consequence that *user workflow*s are often similarly degraded. Contemporary simulator scripts are also typically unstructured in the sense that a biological model is mixed with other code that defines and controls inputs, outputs and simulation configuration [21].

### The User Workflow

The term *user workflow* is employed to describe the sequence of necessary steps typically employed by a person in developing a computational model and employing simulation to generate data for subsequent analysis. In this sense it is a depiction of a sequence of operations, declared as the work of a person or a group of persons [30].

A comprehensive user workflow can be employed to guide the separation of the different aspects of a model by organizing user actions into different categories during model development. The workflow allows distinctions to be made between an object under investigation, the tools used to perform the investigation, and the operations performed during the investigation. It also distinguishes between the results obtained from a single investigation and the method used to define multiple investigations in a series. The user workflow identifies five steps in total (explained in more detail in Results).

### The Ideal User Workflow for Simulations in Neurobiology

As many more data flows can exist than are present in reality, each actual data flow can be considered in the context of a sequence of user actions or workflow. We define an “ideal user workflow” that provides a canonical form of a user workflow specific for neural simulators. In this section, we introduce the set of typical workflows that use CBI simulator architecture applications by describing the ideal user workflow where a user wants to model a biological system. We then briefly mention a second set of workflows that comprise user extensions of the functionality of an implementation of the CBI architecture. Both sets of workflows are presented in a technology and implementation free manner.

A five step outline of an ideal user workflow for the development, implementation, and simulation of a computational model has been identified from the workflow of users of the GENESIS neural simulation platform [31]. Importantly, the workflow explicitly distinguishes between the static structure of a model of the biology (Step 1), the dynamic state of its simulation (Step 3), and the analysis of this dynamic state (Step 4). We also note that this workflow does not specify any particular order for its completion. However, for any given case, meaningful simulation output will only occur with completion of Steps 1–4.

### Step 1: Construct Model

The simulator shell and the graphical user interface (GUI) each provide an interface that interprets user input such that the simulator ‘understands’ different commands and performs the appropriate actions. Simple models can be created directly within the simulator shell by entering a sequence of commands. More complex models are available to the shell from libraries or databases external to the simulator. Shell tools can then be used to explore and check the integrity of a model. Following any necessary or desired changes, a new version of the model can be saved.

### Step 2: Design Experiment

Specific change management tools can be used to make small modification to a model, e.g. to set model parameter values specific to a given simulation. Configuration tools support the definition of the stimulus or activation parameters for a given simulation run or experiment and the output variables to be stored for subsequent analysis by independent software.

### Step 3: Run Simulation

Shell tools can be used to check the state of a given simulation or reset the simulation time step and solved variables to their initial values. After a simulation is run, output values are flushed to raw result storage for subsequent data analysis. The model state can be saved at any simulation time step. This allows it to be imported into a subsequent simulator session for further development and exploration.

### Step 4: Process Output

The validity and location of simulator output is checked prior to data analysis. Output can be analyzed either within the simulator or piped to external applications such as Matlab.

### Step 5: Iterate

A modeling project is established by the introduction of iterators into the user workflow. Iterators close the loop between the output of results and model construction, they include: Automated construction of simulations and batch files, static parameter searching, and active parameter searching using, for example, dynamic clamp technology.

### Principal Concerns

In software engineering, the process of partitioning a program into logical functions that minimize overlap is referred to as a *separation of concerns*. We consider that a principled separation of concerns is a prerequisite for the development of advanced computational modeling techniques in the neurosciences. User *concerns* have a direct influence on the user's experience of an application. Technical concerns have a clear and direct influence on the partitioning of a program into its primary functional blocks, and are also crucial for problem diagnosis and guaranteeing the correct behavior of software. Here, we propose there are two principal concerns that underly the development of modeling software: (1) Separation between data and control, and (2) Separation of biology and mathematics through the use of data-layering.

### Control versus data

All software can be understood in terms of algorithms operating on input data to produce output data. For experimental research, the natural distinction between data and algorithms can be compared to the distinction between the biological system (data to be investigated) and the stimulation paradigm (tools supporting data investigation). For computational research, the distinction between data and algorithms leads to a separation between model (data) and simulation control (control of data flows).

### The requirement for data-layering

It is the capacity to represent a model in terms of biological concepts such as neuron, dendrite, soma, channels, and molecules, that allows a user to clearly relate a model to the original scientific questions. One way to achieve this goal is to separate the high-level biological representation of a model from its low-level mathematical implementation and provide operators that convert between them. This insulates the process of model construction from the computations performed during a simulation.

**Figure 2:**
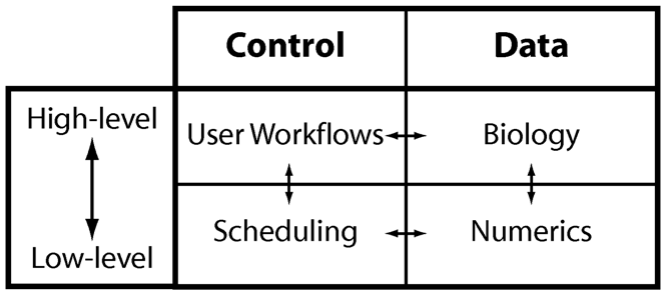
Principle concerns. The four fundamental building blocks of a simulator are distinguished by separating (i) Data from control, and (ii) High level biological concepts from their mathematical implementation. In a federated architecture the only allowed interactions between modules are those indicated by the vertical and horizontal arrows. Diagonal interactions are forbidden as they ultimately lead to interactions that result in the existence of a monolithic software architecture.

The relationships between simulator control and data modules in a federated architecture are symbolized by the horizontal arrows in Figure 2, whereas the relationship between high level biological concepts and their mathematical implementation are symbolized by the vertical arrows. It is the separation of the principle concerns along the horizontal and vertical axes that allows independent modules to be designed. This process underlies the construction of a simulator composed of stand-alone *component*s and forms the basic meta-framework referred to as the CBI architecture.

Importantly, we note that a simulator can be efficiently modularized when only horizontal or vertical interactions are allowed between the modules illustrated in Figure 2. The larger the number of interactions allowed between diagonally located components, the more difficult it becomes to functionally separate simulator components and to maintain and extend the resulting software application. Diagonal interactions are forbidden in the CBI architecture as they foster the mixing of functionality across different levels that ultimately leads to the creation of monolithic software applications.

### Separation of Concerns

Consideration of the principal concerns of data, control, and data layering were used to expand Figure 2 by the separation of concerns principle. This key principle in software engineering states that a given problem involves different kinds of concerns. To cope with complexity, these concerns should be identified and separated [32]. The aim is to achieve engineering quality factors such as robustness, adaptability, maintainability, and reusability. Ultimately, this results in clear model scripts where the biological aspects of a model are separated from the peripheral code that implements a model during a simulation.

In this section we present the outcome of a separation of concerns based on the principal concerns of data- and control-related simulator components introduced above. Initially, the biological and numeric representations and user workflows and scheduling modules are expanded to give the principal functions of the CBI architecture.

**Figure 3:**
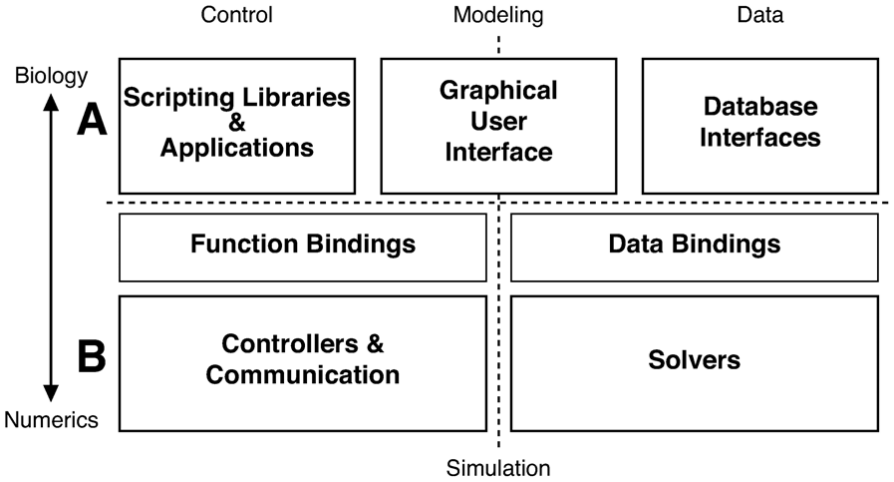
Overview of a federated software architecture. Graphical illustration of the primary functional modules defined for the CBI federated software architecture. Control modules are given to the left and data modules to the right. A. The top layer contains conceptual data and controls representations of the biology of a model. B. The bottom layer contains representations that are numeric and thus close to the hardware. The middle or intermediate layer bridges between the biology and the numerics implemented in a CBI compliant simulator. Importantly, as our separation of concerns shows (see Fig. 2 and text), Control (Scripting Libraries & Applications) and Data (Database Interfaces) modules can interact either directly or via the Graphical User Interface.

Our analysis generated the primary functions of the CBI architecture illustrated in Figure 3. The mechanism identified for separation of model construction from the low level computations performed during a simulation was the addition of a mid-level software layer. This intermediate layer provides function and data bindings between scripting applications and database interfaces, respectively, and the low-level back-ends. Note, this figure maintains the relationships between the four principal concerns identified in Figure 2 by separating high level biological representations (Fig. 3A) and low level mathematical implementation (Fig. 3B), as well as separating control functions from data streams. Note also, the addition of a GUI to connect high level scripting applications with database interfaces.

### User Workflows and Biological Data

The first step in our ideal user workflow involves creating or importing a model. It maps directly to high-level biological representations via a simulator shell or GUI. This interface straddles the Control/Data divide and replaces the upper horizontal arrow connecting the User Workflow and Biology modules in Figure 2. It enables the workflow by assisting either the development of simple cell models from the command line of a simulator shell via the Scripting Libraries & Applications module or the importation of model descriptions via the Database Interfaces module (see Fig. 3).

Step 2 of the ideal user workflow typically requires biological expertise to design an experiment. This includes the definition of constants such as the command voltage of a voltage clamp protocol, delays and duration of a current injection protocol, and model and simulation inputs and outputs.

### Numerics and Scheduling

Step 3 of the ideal user workflow deals with the checking, resetting, and running of a simulation. This is accomplished via the Function Bindings module of the CBI architecture (see Fig. 3).

At a technical level the simulation involves scheduling mathematical operations on, and communication of, the numerical representations of a biological model. This step is indicated by the horizontal arrow between Scheduling and Numerics in Figure 2 and is encompassed by the Controllers & Communication and Solvers modules illustrated in Figure 3.

Elaborate user workflows can stop and restart running simulations and provide new inputs to a model thereby imposing high-level control on the low-level back-ends via the controller.

### Overall Design Objectives

Several important objectives emerged from the separation of concerns and were used to guide the development of our federated approach to the design and development of a neuronal simulation engine. They include: (1) Reduced complexity of software modules when compared to a monolithic system, (2) Simplified documentation of modules in terms of inputs and outputs, (3) Easy incorporation or removal of individual modules as required, (4) Simplified development and testing of modules as stand alone components, and (5) Clear delineation of scope for new module development.

### Structural Overview of the CBI Architecture

The CBI architecture is defined as a modular paradigm that places stand-alone software components into a set of logical relationships. In this sense it defines a modular framework that provides the necessary parts of a neural simulator.

The schema identified by the separation of concerns (see Fig. 2) is expanded in Figure 3 to give the modules that form the building blocks of the CBI architecture. This figure retains the four quadrants of simulator functionality identified by our separation of concerns. It includes the notions of low-level data for numerics and high-level representations for biology, as well as separation between data and control (Fig. 3 indicated by horizontal and vertical dashed lines, respectively).

We refer to the CBI architecture as being ‘federated’ as it extends the modular approach associated with the development of single applications to the functional integration of otherwise independent applications. Federation aims to provide a unified interface to diverse applications and mask from the user the differences, idiosyncrasies, and implementations of the underlying applications and data sources (see www-128.ibm.com/developerworks/db2/library/techarticle/0203haas/0203haas.html). In doing so, federation provides transparency, heterogeneity, a high degree of function, autonomy for the underlying federated sources, extensibility, openness, and the possibility of highly optimized performance. Here, extensibility is defined as a system design principle where an implementation takes into consideration future developments. An extendible system is one that includes mechanisms for expanding or enhancing the system with new capabilities without having to make major changes to the system infrastructure.

Ideally, it makes the underlying applications look like a single system to the user. Enhanced application interoperability is achieved through the use of flexible high-level *scripting languages* to support diverse workflows and low-level *application programmer interfaces* and *application binary interfaces* for performance.

In summary, the CBI architecture provides a template for software development that, at its core, contains a simulator. Additionally, the modularity and layering of the architecture simplifies connection to independent applications indirectly related to model construction and instantiation and the display and analysis of simulation output. Figure 3 illustrates the various modules of the CBI architecture.

### The High Level User Interface Layer

Modules in the top level of the CBI architecture (Fig. 3A) provide user accessible interfaces to simulator functionality through a Graphical User Interface. Model data are controlled by the Database Interfaces, whereas, simulations are controlled by Scripting Libraries & Applications. The various modules and submodules located in this layer can be instantiated by a simulator on an as-needed basis. This layer supports the user interfaces for the first three steps in the ideal user workflow, including: (1) Construct Model, (2) Design Experiment, and (3) Run Simulation.

### The Graphical User Interface

**Figure 4:**
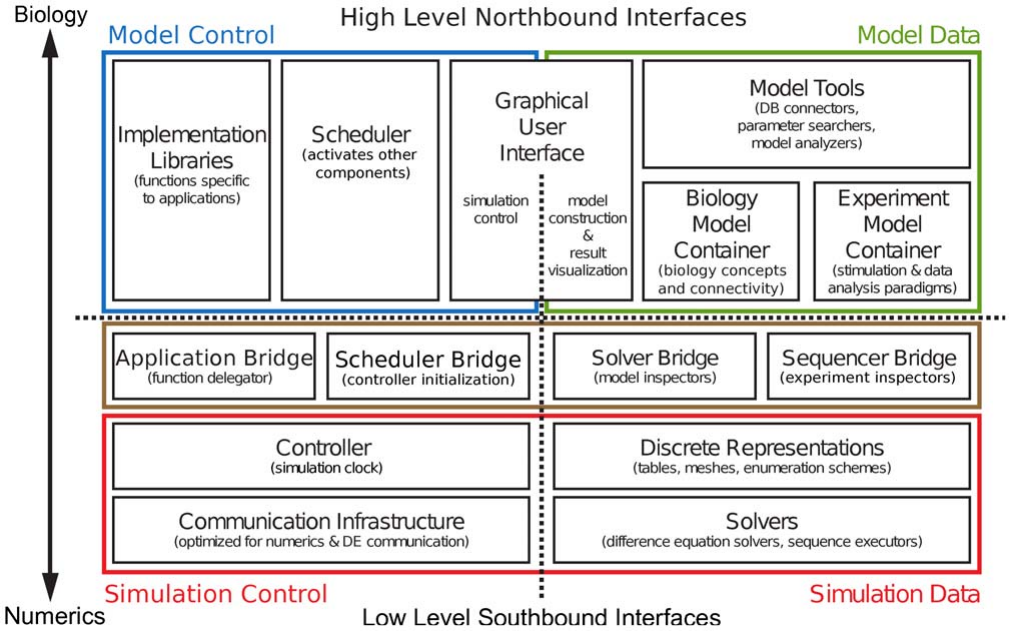
Detailed view of the Computational Biology Initiative federated software architecture. Illustration of the functional modules that is closer to an implementation of the CBI architecture. It illustrates the relationships of sub-modules within each of the primary functional modules given in Figure 3. North bound interfaces group and conceptualize the details of the modules and interact with south bound interfaces of higher level modules. Steps 1–3 of the ideal user workflow induce data flows between the software modules of the CBI architecture. This results in data cycling between the upper layers (blue and green boxes) and lower layers (red box). Ultimately, the two layers team to implement a single simulation. By design, any type of model including multi-scale models will exhibit this data cycle. See text for explanation and details.

Based on the distinction between data and control, the GUI comprises two submodules (illustrated in Fig. 4). One is related to model data and incorporates model construction and visualization of simulation results, the other is related to simulation control.

#### Model Construction and Result Visualization

The model construction functionality of the GUI allows a user to define a model in terms of the biological properties supported by the modeling component (the Biology Model Container, described below). It also connects to other modeling components to provide a GUI for database connectors in the module Model Tools and the Biological and Experimental Model Containers. This GUI can also be used to inspect or alter a model and instruct the modeling component to save the model back to disk as a conceptual representation available for later use.

The result visualization functionality of the GUI provides different views of a model and supports the possible workflows between them. It can be divided into generic and application specific parts. The generic parts are those commonly provided by external tools such as GNUplot (http://www.gnuplot.info/), Matlab (http://www.mathworks.com/), or Grace (http://plasma-gate.weizmann.ac.il/Grace/) for display of the temporal evolution of variables. Examples of the application specific parts include, user specified visualization of neuron morphology via morphologically related color coding of variables solved during a simulation, axons showing propagating action potentials, and higher level visualizations of network behavior [33], [34].

#### Simulation Control

Simulation control comprises actions such as the starting and stopping of a single simulation while a browser provides access to sets of related simulations and results that can be explored with the Result Visualizer. Various buttons and dialog boxes allow interaction with the Experiment Model Container to configure protocol specific inputs and parameters (see below).

### Database Interfaces

There are three primary submodules within the Database Interfaces module. They are: (1) Model Tools, which provides functionality for connecting to databases, parameter searches, and model analysis, (2) The Biology Model Container, which allows a user to define a model in terms of biological properties such as spine, morphology, circuits and their connectivity, and (3) The Experiment Model Container, which enables the definition of an experiment in terms of actions taken on a biological model.

#### Model Tools

Some model tools may already incorporate a good GUI for model construction (for a typical example see [35]). The difference with these tools is that in the CBI architecture this part of the GUI does not export a model in a simulator specific language. Instead, it interacts directly with the Model Containers via a low-level *application binary interface* (ABI). (Note: For simplicity, where appropriate, we collectively refer to the Biology Model Container and the Experiment Model Container as the Model Containers.) The ABI provides a direct coupling between the implementation technologies of the different software components, creates a much tighter loop between the GUI, Simulation Control, and lower level back-end functions (described below); and provides a richer interactive user-experience.

#### The Biology Model Container

This module stores three different versions of a model. (1) A biological representation, available for inspection by other software modules, e.g. a model visualizer, (2) A conceptual representation that can be regarded as an enumeration of biological concepts and their relationships. It can contain algorithm names and parameters that specify how to build the model and can be exported and stored on a file system, and (3) A fully expanded mathematical representation, generated by algorithms referenced from the conceptual representation, which, if mathematically complete, can be simulated. However, if a parameter is missing, e.g. the axial resistance in a neuronal morphology, the model is still useful for inspection and visual validation by the Model Construction GUI. Note that most current simulators do not allow incomplete representations of a model.

The translation between conceptual and mathematical representations involves separate tasks, (1) Linearization of the hierarchical structure of the biological model for use by the solvers and (2) Translation of model connectivity to connectivity between these solvers. We now briefly expand on these tasks.

If biological components in a model are assumed to function as a single biophysical unit they can be grouped, e.g. a spine and a dendrite, an axon hillock and the soma, or a population of calcium or potassium channels. For a mathematical solver however, these groups must be converted to a set of equations at the same level as other equations of the same type and independent of the hierarchical structure of the biological component. This requires translation of the biology of a user defined model to the necessary flat stream of elements.

In a biological representation of a network model there are hierarchical projections, each containing their own sub-projections and connections. However, during a simulation the different back-ends of the CBI architecture are only connected by a set of serial communication ports. It is the Connectivity Translator that translates the connectivity between the components of a biological model to that of the connectivity between low-level back-ends (described below). This requires the Biology Model Container to also act as a model component identification system: Given the identifier of a biological component in the mathematical representation, the Biology Model Container must be able to translate it into an identifier for a mathematical variable that can be used by a solver.

By implementing biological and connectivity translation, the Model Container decouples the physical implementation of the low-level back-ends from the biological representation of a model and the way the user sees the model. The advantages of this approach are (1) The implementation complexity of the mathematical solvers is greatly reduced as they have only to deal with numerics and (2) It enables a more intuitive and user-friendly representation of a model.

Depending on the simulator application domain, an implementation may focus on one of the translation functions or both. For instance a network simulator such as NEST provides better support for connectivity translation [36], while a single neuron simulator typically provides better support for biological concept translation. The GENESIS and NEURON simulators support both connectivity translation and biological concept translation. However, their translation functionality is essentially one-to-one such that the representation of the connectivity of the biological model exposes the structure of the mathematical equations. This results from a violation of the data-layering principles identified by our separation of concerns. One consequence is, the user becomes responsible for managing network connectivity at the mathematical level during construction of network models.

#### The Experiment Model Container

This stores a model of a stimulus paradigm and desired output parameters and defines and stores a hierarchical sequence of stimulus-related actions (e.g. start and stop time of a current injection) and their dependencies.

### Scripting Libraries & Applications

This module contains three submodules, (1) Implementation Libraries containing functions specific to a given application, (2) Scripting Libraries that provide scripts to control a specific series of research and tutorial simulations, and (3) The Scheduler, which combines generic aspects of these scripts to instantiate the simulation.

#### Implementation Libraries

Each library has a defined focus, e.g. the investigation of a synaptic learning rule applied to a given neuron, or a library of stimulus paradigms representing in-vitro and in-vivo conditions for a cerebellar Purkinje cell model [37], [38]. The library implementation typically occurs for a set of research or tutorial simulations. It may also implement ‘glue’ functions to connect to external libraries for other activities such as result analysis, e.g. via the GNU Scientific Library (http://www.gnu.org/s/gsl/), the Perl Data Language (http://pdl.perl.org/), and the Scientific Library for Python (http://sourceforge.net/projects/scipy/).

#### Applications

A typical example of an application is a set of simulator scripts that implement a model tutorial (e.g. [39]).

#### Scheduler

For stand-alone software components such as the Model Container and the low-level back-ends to be useful, they must be ‘glued’ together and activated correctly, such that they can work together in co-ordination on a single simulation. This is exactly what the Scheduler does. It typically exploits the sophistication of modern scripting languages to load software components on demand and initialize and activate them from a configuration file. The Scheduler does not have any computational load and its implementation is both simple and highly configurable. It can also provide a script-based interface for interactive user control or the implementation of more sophisticated user-workflows.

### The Low Level Back-End Layer

Modules in the lowest level of the CBI architecture (Fig. 3B) provide, (1) Solvers that employ specific algorithms for the solution of numerical equations. They include difference equation solvers and sequence executors that can discretize and tabulate fixed mathematical functions according to a user settable accuracy and (2) Controllers (such as the simulation clock) and Communication Infrastructures optimized for discrete events and numerics. Collectively these modules implement the run-time environment of a simulator.

### Mathematical Solvers

Mathematical solvers apply numerical techniques such as Runge-Kutta [40] or Crank-Nicolson [41] to solve systems of equations. Dedicated data structures can be employed that adapt these methods for computational neuroscience. Mathematical solvers include compartmental, kinetic pathway and Monte Carlo solvers, and in general any low-level back-end at the numerical or hardware level. Because of their numerical nature, some solvers can easily be extended to do the necessary discretization of a continuous mathematical equation (at a user settable accuracy). The generation of channel conductance tables is a common example. Other examples are mesh generation for Monte Carlo solvers and compartmentalization of a neuronal morphology.

The **Command Sequence Executors** provide a physical implementation of an experimental protocol *in computo*. A stimulation protocol stored by the Experiment Model Container originates a Command Sequence Executor and the stimulus events associated with the given protocol propagate to the input port of a solver at a given time step. Note, connectivity between command executors and numerical solvers is provided by the connectivity translation function of the Model Containers.

#### Discrete Representations

Existing monolithic simulators such as NEURON and GENESIS 2 currently require the user to explicitly code scripts to avoid duplication of one-off data. In contrast with this GENESIS 3, in compliance with the CBI architecture, automatically detects duplicate parameterization of variables such as channel gate kinetics and dynamically shares their tabulated form as necessary. Solver generated discretisations that form the numerical representation of a model are internally published for reuse by other solvers in a process that is invisible to users, e.g. dendritic morphology meshes and Hines enumeration of compartmentalized morphology. Annotation of the representation is necessary, e.g. annotation with the time step used to generate the representation. The reuse of such representations minimizes the memory requirements of large simulations.

### Controllers & Communication

This module contains several submodules related to the low-level control of a simulation.

#### Communication Infrastructure

The Communication Infrastructure establishes run-time communication between different solvers and input and output elements working on the same model during a simulation. It can be optimized for either discrete event communication or communication of array based numerical data. There is a differential implementation for serial as opposed to parallel hardware. As it can be part of the run-time system during a simulation, the Communication Infrastructure can have a significant impact on run-time performance. Depending upon the simulation and hardware involved, Communication Infrastructure deals with issues of (1) Parallelization, such as whether solvers are collocated on the same CPU and whether this is made transparent to the solver implementation, (2) Efficiency of the infrastructure for communicating neighboring lists of values, e.g. all the membrane potentials of a compartmentalized neuronal morphology, and (3) Efficient storage and distribution of discrete events used for action potential generation and axonal propagation.

#### Controller

The Controller maintains the operation of all simulator functions. It contains core functions such as the global simulation time clock and the functions that start and stop the advance of simulation time. The main function of the Controller is to schedule and synchronize the back-ends that participate in the simulation by requesting the numerical level to update its internal states. To leverage the operation of sophisticated solvers and enhance their maintainability, the Controller can also implement facilitatory functions for arithmetic control such as floating point exceptions.

### The Intermediate Layer

The intermediate layer comprises the bridges for function and data bindings that assist with the vertical flow of information during the translation of biological concepts into numerical equations.

### Data Bindings

Data bindings translate the expanded representation of a model into data structures that are specific for one type of solver. They interface the Biology Model Container with the numerical back-end and allow a natural and efficient code for the solution of the numerical equations. Note that a data binding implementation can choose to bind a solver to only one of the two translation functions of the Biology Model Container. For example, a single neuron solver only needs to access the task of biological model translation.

The most important characteristic of a data binding is that it contains no specific algorithms. Instead, it provides a one-to-one mapping of selected data extracted from the Biology Model Container. The data selection can omit certain properties of the biological model, i.e. geometrical coordinates are commonly not used for the numerical solution of an equation.

A library of functions in the Biology Model Container assists in the translation of values from a continuous mathematical domain to a numerical domain. Examples include, the scaling of a conductance density to a maximal conductance or the scaling of the membrane capacitance density to the actual membrane capacitance with respect to the surface area of a given compartment. Superficially, this seems easy, although it is not always the case, e.g. when spines are present in a model the capacitance density must be scaled to account for the additional spine surface area, while for a conductance density the spine surface area is commonly not taken into account [38].

Other data bindings translate a sequence of stimulus actions and output definitions to commands of a Command Sequence Executor. They decouple the sequencer back-end implementation from the Experiment Model Container such that the commands from complex stimulus paradigms can be correctly sorted on both non-parallel and parallel architectures.

### Function Bindings

The function bindings connect user actions with functions of the Controller such as to start and stop a simulation. They also translate application specific control statements to events or actions for the Controller.

### A Short CBI Architecture Implementation Guide

Software implementations are formal structures that map user requirements to technological solutions. However, an application can only meet those requirements when this technological mapping exists. In other words, it is the available technology that defines the scope and boundaries of the user-workflows that can be implemented in software solutions. As a consequence the development of entirely new software architectures is best started from the lowest layer and constructed upwards to implement user-workflows and satisfy user requirements.

The lowest layer of the CBI architecture comprises numerical solvers, thus implementation of a CBI compliant simulator can start with the implementation of a numerical solver. Firstly, source code files can be populated with the mathematical functions that implement the solutions of the targeted equations. Secondly, functions are required for interaction with the Controller module. After these two steps, the new solver is useful for the simulation of simple models. Thirdly, functions are required for communication with other solver software components. Implementation of an interface with a Biology Model Container makes the solver available for simulation of more realistic models. Finally, connecting with an Experiment Model Container and Scripting Libraries allows applications such as tutorials and research projects to be developed.

### Behavioural View of the CBI Architecture

Figure 4 provides an expanded view of Figure 3 and illustrates in more detail the structural relationships between the different modules and sub-modules that comprise the CBI architecture. We introduced their functionality above. The behavior of the CBI architecture is defined by the functional and dynamic connectivity provided by these individual modules. We now describe this behavior within the context of our ideal user workflow.

#### Data-flows in the CBI Architecture

A (G)UI translates user actions into a family of events that propagate to other components of a software architecture, impact the internal states of these components, and direct the data flows between them. In Step 1 (Construct Model) and 2 (Design Experiment) of the ideal user workflow, users combine models and experimental data that are stored in files and databases. In Step 3 (Run Simulation) the back-ends, such as the numerical Solvers and the Communication Infrastructure, perform the calculations of a simulation, then save the output back to files and databases. When combined, these steps of the ideal user workflow imply a cyclic data flow from files and databases to the back-ends. Here we explain how user actions and data flows relate to one another in the CBI architecture and define the overall behavior of an implemented software system.

In Step 1 of the ideal user workflow, the GUI is opened and a cell model is selected from a database listing of available models. Internally, model selection is translated into an event that instructs the Biology Model Container to load a selected model from a database and store it in memory using data structures for efficient storage and retrieval by other modules. During initial inquiry, a user may typically be interested in derived model parameters such as the total surface area of a neuron, with and without spine correction, while a sophisticated GUI can also present a table of the channels employed in the model along with their conductance densities and reversal potentials. More dedicated queries related to specific brain areas or neuron types are supported by the Scripting Libraries & Applications module.

A typical example of Step 2 of the ideal user workflow is the design of an experiment that applies current injection pulses to a neuron's soma and defines simulation output as the somatic membrane potential and somatic transmembrane currents. The Experiment Model Container stores the definition of the current pulse amplitude and duration, which is translated by a Sequencer Bridge to a sequence of simulation-time events prior to the start of a stimulation. These events are then executed by the Command Sequence Executor during a simulation.

Running the simulation in Step 3 of the ideal user workflow starts with the Biology Model Container examining a stored model to determine model time constants or other parameters that are relevant for the accuracy of a numerical simulation. The Solvers then fill their data structures with parameter values optimized for simulation, for instance a Crank-Nicolson solver can multiply the membrane capacitance with the time step during this initialization phase instead of at every simulation time step [42]. For this, the memory image of a model must first be expanded into a representation that includes the mathematical equations and parameters relevant to the given simulation but, for instance, does not include the spatial layout of segments as this is not required by the Solvers. (Note: ‘Segment’ is a high level term employed to describe different parts of the biological model of a dendritic morphology. The equivalent low level (computational) term is ‘compartment’. It refers to the numerical representation of a segment.) This behavior is different from that of existing simulators, e.g. GENESIS 2. Such simulators do not make an explicit distinction between internal data structures for model representation and data structures for computation. Consequently, they often generate redundant data during the initialization of a simulation.

In network simulations the Solvers employ the Connectivity Translator to initialize its simulation-time communication data structures and to connect to the Communication Infrastructure.

When a user instructs the simulator to start a simulation, for instance by pushing a button in a GUI, the Controller generates a list of instantiated Solvers. It then advances the simulation clock and requests each Solver to update its internal state. The Communication Infrastructure connects the Solvers for efficient communication of the solved variables. Solvers that were configured for output, save results to a file. When the simulation finishes, either by user action or following a preset simulation period, output buffers are flushed to disk.

The independence of the Solvers in the CBI architecture not only allows for better optimized implementations, but also enables additional simulator functionality such as serialization of the model state to a file. This allows a simulation to be resumed at a later time and reduces the total simulation time of a complex model if it requires a calibration phase prior to the application of an experimental protocol.

As a result of Steps 1–3 of the ideal user workflow, data flows both through and between software components that conform to a CBI architecture: the data cycles between databases and files, and back-end Solvers. Here we have described this cycle for a single neuron model and, as we briefly noted, it also occurs for network models. By design any type of model including multi-scale models will exhibit this data cycle.

In Steps 4 and 5, the availability of any model data from the Model Container and the functionality of Scripting Libraries & Applications can connect the CBI architecture with external tools while maintaining the integrity of the separation of concerns. Scripting Libraries & Applications connect to external tools such as Matlab to implement output analysis. They also allow simulation output data to be combined with the model parameters and structure available in the Biology Model Container to implement Step 5 of our ideal user workflow, for instance to provide automated script generators for the generation of batch simulations. Importantly, at this stage of the workflow, the back-ends of the CBI architecture (indicated below the dotted horizontal line in Figures 3 and 4) are unavailable. Once Step 3 is completed, all the data of the dynamic state of the model are available through databases and files. Consequently, there is no requirement to query the software components that deal with numerical data. This ultimately prevents the implementation of diagonal interactions in the software and the creation of a monolithic simulator.

## Discussion

Considerable experience with both user and technical concerns related to simulator functionality and efficiency has been gained from over twenty years of following user requirements during the development of bottom up models of neurons and neural circuits within the framework of the GENESIS software platform. This has allowed us to identify an ideal user workflow. We employed this workflow to constrain the separation of concerns analysis that lies at the heart of the new software architecture we describe here.

By analyzing and decomposing a software system into separate functions, it becomes straightforward to define the individual software modules of the architecture. Clear delineation of individual components in a modular software architecture allows interfaces and their accompanying behaviors to be defined and the communication between software layers to be specified.

We employed the results of our separation of concerns analysis to develop the software architecture for a next generation neural simulator. The resulting CBI architecture provides three significant advantages for software development: (1) Modules can be run separately on different machines. For example, the GUI and modeling environment might run locally, while the simulator is run elsewhere either serially or in parallel on more powerful machines. (2) Decomposition of an application into multiple software components allows reuse and extension of individual modules. This clearly facilitates model development and research progress. (3) Individual components can be independently updated, enhanced, or replaced when needed, thus the life cycle of a modular architecture is less complicated than that of a non-scalable application.

Our approach has the advantage that the mathematical and optimization internals of a solver need not be exposed to a user. The resulting biological and numeric layers can further be individually optimized to provide significant enhancements in overall system performance. For low-level data and control, such optimizations consist of running simulations more rapidly, e.g. by achieving high cache hit ratios [42] and parallel implementation [43]. For high-level data and control, optimization involves increased support for biological concepts and usability by employing both domain specific and scripting languages, as opposed to low-level languages [44]. This improves support for user-workflows and gives a richer user-experience [45].

### Comparison of the CBI Architecture to Software Industry Standards

The CBI Architecture is based on a three-tier software architecture, a widely used commercial client-server paradigm [46]. This paradigm has the dual advantages that servers can be shared by users and software on both the client and server is easily upgraded. Service-oriented architecture (SOA) is a widespread commercial implementation of a three-tier architecture where service based information exchange, reusability, and composability defines a software federation for an application by uniting resources while maintaining their individual autonomy and self-governance [47].

### User Experience

Dedicated industry standards have been developed for SOAs. They allow routine implementation of business logic with an optimal user-experience. (For example, the Business Process Execution Language–BPEL–was approved by the industry in 2007. See http://en.wikipedia.org/wiki/Business_Process_Execution_Language.) SOAs often store control tables for meta-data describing user workflows [48]. In this way, control software is replaced with software for data management and the technical implementation becomes focused on data management. Consequently, SOA code development is highly focused on database queries, result integration, and presentation. This provides a sharp contrast to preliminary developments in computational neuroscience for the standards of educational tutorials, research project frameworks, and declarative neuronal modeling standards such SED-ML [49]. Control, as defined for the CBI architecture, is not a major aspect of SOA implementations. As a result, SOAs are architectured using data-centric and technical concerns such as database transactions and data routing, logging and debugging rather than the principle concerns of data and control that we employ here.

### Software Structure and Run-time Parallellization

In three-tier architectures it is not uncommon for the second tier, comprising so called middle-ware, to be distributed over multiple hardware servers. However, any parallel structure is specific to a given business application and the business logic is fixed during the application design phase. This is not usually the case for neuroscience simulators which often use the CPU load of the mathematical model to calculate an ideal partitioning for each simulation. This makes the parallel structure potentially different for each simulation run [50]. In other words, the static parallel structure of a three-tier application can be contrasted with the dynamic parallel structure of a CBI compliant simulator where the parallel structure of the run-time environment may depend on model structure and simulation number [14], [51].

It is for this reason that an optimal implementation of a CBI compliant simulator includes functionality to automatically partition a model and to transparently distribute simulations within a parallel computing environment [43], [50], [52].

### Differences in Backend Focus

Importantly, the implementation of business logic as control tables results in the back-ends of SOAs consisting of database engines that typically embed specialized algorithms and data structures for information query optimization and performance. In contrast with SOAs, the CBI architecture provides explicit support for control functions throughout all layers of a CBI compliant simulator through the Scripting Libraries and Communication modules.

### CBI Simulator Extension

When compared to the current generation of simulators, simulator extension has a greatly enhanced meaning for a CBI compliant simulator. Firstly, simulator extensions can be implemented as modifications of and additions to existing software components. As an example it can be relatively easy to enhance a simulation controller such that it prints the simulation time to the screen. Similarly, it is easy to enhance an existing compartmental solver to compute the average membrane potential of a defined section of a cell or its dendrites.

A second class of simulator extension is the addition of new software components. For instance, to connect the simulator to a morphology database and a channel model database two database connectors can be written. They convert the database formats to *application programming interface* (API) calls to the Biology Model Container. Simple extension of this module allows it be used for analysis of morphological characteristics and for quantification of model components.

These different types of extensions stem from the modularity of the CBI architecture. Current simulators typically only support a restricted set of extensions. For example, extending the GENESIS 2 simulator with new solvers is not possible because its basic architecture is not suitably modularized.

### CBI Simulator Interoperability

Historically, the emergence of monolithic neuronal software has led to simulator interoperability in computational neuroscience being addressed as Type 1 interoperability between stand-alone applications. It has also been proposed that Type 2 interoperability can be achieved through the development of declarative markup languages such as NeuroML and NineML [21].

A CBI compliant simulator easily supports Type 1 interoperability through implementation of the appropriate database interface software component. Such an interface maps the concepts of declarative languages to concepts supported by the Biology Model Container, which can also be used to connect the simulator to external databases that provide support for these declarative languages.

Type 2 interoperability has recently been implemented as the capability to communicate simulation data at run-time between different simulators using procedural paradigms[24], [26]. However, this approach requires the modeler to understand some of the internals of the model, the simulator, and the implementation of the procedural paradigm. While monolithic software applications are typically too complicated to extend, this additional layer of complexity makes the modeler's task next to impossible.

Ultimately, the CBI architecture moves interoperability problems from a context of communication between large monolithic applications to communication between well defined and simpler software components. Here, it is the communication infrastructure of the CBI architecture that addresses the run-time communication of simulation data. It is within the context of the CBI architecture, that the support of both Type 1 and 2 interoperability becomes a practical possibility.

### The Ideal User Workflow Revisited

The steps of the ideal user workflow can be categorized as user-workflow oriented and data-oriented. We briefly showed above the relationships between the ideal user workflow and the CBI software architecture from this viewpoint.

The steps for model construction and experiment design naturally correspond to the configuration definitions of the Biology Model Container and the Experiment Model Container, respectively. The integration of these two steps, provides a single simulation that can be run by the mathematical Solvers, the Communication framework, and the Scheduler. Each of these components needs appropriate peripheral configuration. The methods used to analyze the results are specific to the scientific question at hand, and can be configured in the component Scripting Libraries & Applications. When combined, these configuration definitions determine one run of a simulation.

The separation of the steps for biological model construction and experiment design allows the modeler to build separate libraries of biological models and experimental protocols. In principle this allows any model to be combined with any appropriate experimental protocol without the requirement that the protocol was specifically designed for the given model. The automation of this process would be useful to identify the strengths, weaknesses, or deficits of a specific model.

Finally the Iterate step defines the differences in configuration between the simulations belonging to the same simulation project, research project, educational tutorial or scientific publication. It is with this step that the ideal user workflow is integrated with the scientific workflow.

### Structuring Scientific Communication

In the scientific workflow a mental model of a biological system translates through a conceptual model to a mathematical representation and a low-level software implementation. This process takes many intermediate representations that are determined by the different choices made by an investigator. Examples include, the value of a parameter, such as the maximal electrotonic length of a compartment, or the algorithm chosen for the numerical methods. Sometimes these choices are predicated on the empirical phenomenology of neuroscience. A good example is the hypothesis formulated by Hodgkin and Huxley that the activity of transmembrane ion channels is controlled by biophysical gates [53].

The sequence of such choices describes a stepwise translation of a high-level mental model into a computer implementation. This translation connects mental models with their implementation directly and provides a model with a richer structure and meaning than its purely mathematical counterpart. From this viewpoint, through its implementation, every model is intimately connected with the simulator used to run it. We note that it is important to make this connection more explicit than is currently the case [54].

If we refer to the choices made during translation of a mental model to its computer implementation as model structure, and to the parameters as model quantities, then a model's history is defined by the history of its structure and quantities. From this perspective, a modeling environment becomes a tool useful for formally tracking advances in the scientific understanding of a biological system, expressed as changes made to a model. It is in this way that the structure and quantities of a model document advances in the formal scientific understanding of a given biological system. Ultimately, the user workflow integrates a simulator into the scientific workflow such that any CBI compliant simulator provides direct support for scientific publication and communication.

## Materials and Methods

In this section we define the elements of our “workflow philosophy”. It is this workflow that guides development of the CBI architecture. It is presented as a glossary of the technical terms, typically specialized for computer science, that are commonly employed in the description and discussion of the architecture. It is followed by a brief technical glossary of terms related to software development.

### The Workflow Philosophy

Monolithic software and *federated software*, described below, are the two extremes of a continuum. The philosophical goal of the CBI architecture is to move simulator design away from the extreme of monolithic software towards the extreme of federated software.

#### Monolithic Software

A *monolithic software* application is one in which the user interface, data access code, and computational algorithms are combined into a single application where the smallest software component is the whole application. It is typically comprised of a set of tightly coupled functions where the modification or extension of one function requires either corresponding alterations throughout or intimate knowledge of the rest of the software code to avoid breaking existing software functionality.

In the past neural simulators were developed or strictly supervised by a single programmer, a situation that typically led to monolithic applications [7]. As a consequence, the functionality offered by simulation software and the scientific problems that could be addressed were inherently limited in scope by the capacity of a single person to manage the required software project [55].

#### Federated Software

As an alternative to monolithic software, *federated software* design allows different individuals to work in parallel on the same software application [56]. In particular, federated software supports the scientific enterprise in general and scientific research in particular, by allowing individual scientists to work on software dedicated to their own research projects.

#### Concerns

One dictionary definition of *concern* is “a matter for consideration” [57]. More specifically for software, the concerns for a system are defined as “… those interests which pertain to the system's development, its operation or any other aspects that are critical or otherwise important …” [58]. A more general definition is “any matter of interest in a software system” [59]. On this basis, concerns are considered to be fundamentally conceptual. They include, but are not limited to, for example, generality, independence, appropriateness, completeness, and ease of use [60]. Further, considerations in the typing of concerns may include: Logical versus physical concerns and simple concerns versus concern groups, also kinds of access, consistency and integrity, and extensibility and stability [59]. To this list we can add interoperability.

Concerns often penetrate into the different layers of a software architecture. Such concerns are called ‘cross-cutting’ concerns, and, because of this property, are hard to implement and maintain. Often special technology is required to implement this type of concern properly.

#### Separation of Concerns

The *separation of concerns* is an important general design principle in software engineering [61], [62]. It aims to control the complexity of programs that are continually elaborated. In summary, it promotes the separation of different interests in a problem, solving them separately without requiring detailed knowledge of the other parts, and finally combining them into one result. In practice, this principle actually corresponds to finding the right decomposition or modularization of a problem. The aim is to design systems that allow different functionalities to be optimized independently. Ultimately, a competent separation of concerns should make it easier to understand, design, and manage complex interdependent software systems.

#### Module versus Component

Modular programming aims to create software composed of separate interchangeable parts identified by a separation of concerns. This improves software maintainability by enforcing logical boundaries between software modules. We refer to each independent part of the CBI architecture as a *module* which may contain independent submodules, whereas, following implementation a module is referred to as a *component*, which may also contain subcomponents. These components communicate with each other by interfaces. An important attribute of a component is that one component can replace another without disabling the system within which it operates. This greatly simplifies and encourages software reusability. It is important to note that a component can only exist within a well defined framework such as that provided by a software architecture (see below).

As an example of the way we distinguish between modules and components, the GUI module in the CBI architecture contains independent submodules, whereas, the GUI component of a simulator implemented in compliance with the CBI architecture contains independent subcomponents dealing with simulation control, the model, and results (see Fig. 3).

#### API and ABI

An *application programming interface* (API) is an interface implemented by a software program that enables it to interact with other software. It facilitates interaction between different software programs. An API provides a library to be used directly via simple function calls.

An *application binary interface* (ABI) describes the low-level details of an interface between an application (or any type of) program and another application. ABIs cover details such as data type, size, and alignment, calling conventions which control how functions' arguments are passed and return values retrieved and sometimes the binary format of object files and program libraries.

A complete ABI allows a program from one operating environment supporting that ABI to run without modifications on any other such system, provided that the necessary run-time prerequisites such as shared libraries are fulfilled. For an example, see the Intel Binary Compatibility Standard (http://www.everything2.com/index.pl?node=iBCS).

An ABI should not be confused with an API which defines a library of routines to call, data structures to manipulate, and/or object classes to use in the construction of an application using that particular (often language specific) API.

Both APIs and ABIs play a crucial role in the implementation of a federated software platform.

#### Scripting Languages

With an increasing requirement for software integration, the use of *scripting languages* is providing an important programming paradigm. Low-level system programming languages are well suited for building functional software components where there is a requirement for computing speed, whereas, high-level scripting languages are well suited for binding software components to build applications where the complexity is in the connections. This division of labor provides a natural framework for reusability. When well-defined interfaces exist between components and scripts, software reuse becomes easy. In this sense scripting and system programming are symbiotic. Used together, they produce programming environments of exceptional power where applications can be developed up to an order of magnitude more rapidly than when a system programming language alone is used.

#### Software Architecture

As the last step of our workflow philosophy, a *software architecture* defines a framework that logically organizes a collection of modules where each module implements one or more concerns, but no concern is identified with more than one module. When modules are implemented as software components and bound together with scripting languages, the components form an application.
